# The Scientific Uses of Very High Towers

**Published:** 1890-01

**Authors:** 


### The Scientific Uses of Very High Towers.

It appears that we are really going to have in London a second, revised and enlarged edition of Eiffel's Tower. Probably the first object of most promoters of such a tower would be to make it pay; but there are other uses a lofty structure of this kind might be applied to, and it would undoubtedly afford valuable means of scientific observation, especially of the meteorological character.

Mr. Glaisher has gone up in a balloon to investigate the state of the atmosphere at different elevations. The proposed tower would allow of an inquiry into the atmospheric conditions up to 1,200 feet. From such a height we should look down upon the hills of Wimbledon and Norwood; and when the Thames Valley and surrounding hills have disappeard in fogland, we should propably, on our ærial station, enjoy a genial warm sunshine in a blue sky, while gazing upon a vast sea of fog, from which the towers of the Crystal Palace would peep out like two light-houses. On our way down there would be endless observations to make on the changes of temperature, or, to speak more to the point, on the absorption of solar heat by the fog stratum. Now that we are able, thanks to Sir G. Stokes' and Mr. Jordan's sunshine recorders, to obtain permanent tracings showing the length of time the sun is shining or obscured by clouds, such an instrument at the summit and at the foot of the proposed tower would not fail to give very interesting records in comparison with each other.

Instruments for measuring the heat of the sun's rays, such as black bulb thermometers *in vacuo*, and actinometers, disposed at the top and bottom of the tower would also give very interesting readings, the instrument at the highest station, probably showing an excess of the sun's heat over the other, because of the atmospheric humidity intervening between the earth's immediate surface and an altitude of 1,200 feet. Thermometers disposed at various heights, and properly screened from radiation, would show the temperature of the atmosphere at various levels, giving this curious result, that the sun would be warmer at the top than at the bottom, but, in the absence of sun, the air would be colder at the top.

At night the distribution of the temperature in the atmosphere is known to be the reverse of the daytime. On a fine clear night the temperature will be found higher at 1,000 feet than it is on the earth, while on a cloudy night it will be much the same at both stations; but we have much to learn on the laws of terrestrial radiation, and the proposed tower would be very useful in that respect.

Of course an observer would not be expected to race day and night up and down a tower 1,200 feet high, to make observations and read thermometers; this would be too great, and indeed an unnecessary devotion to science. The whole thing can be done from below in a comfortable building by means of Siemens' electric thermometers. Such an instrument has been used for some time, and is now at work at the Lincoln Cathedral, giving correct thermometer readings on the earth's surface from a height of 270 feet.

Besides observations for temperature at various altitudes, the state of humidity of the atmosphere, a condition greatly concerned in the formation of fogs, could be readily examined, dry and wet bulb thermometers disposed at various heights and in electrical connection with the station below would yield interesting observations, and assist materially, in conjunction with the barometer, in forecasting the weather.

The force and direction of the winds at various altitudes are subjects for meteorological inquiry of much interest and importance; there are eddies in the atmosphere, spirals and upward currents, of which little is known, and a tower such as that now contemplated would allow of very interesting observations to be made on winds. We may also venture to suggest that the information thus obtained would have an important practical bearing in an engineering point of view, as showing the strength of the wind buildings may have to withstand at great heights.

The measurement of rainfall, made simultaneously at 1,200 feet and on the ground, would add to our knowledge of the laws of rainfall; it would be found that less rain falls at the top than at the bottom. The connection of this phonomenon with atmospheric humidity and temperature would be very interesting to investigate. But fancy being in the track of a thunderstorm in such a lofty, mid-air station, and perhaps enjoying the sight of lightning not above, but below, with deafening peals of thunder and rain forming underfoot; hail may be heard rattling from one cloud to another, or brushing sidewise into the face. This, indeed, would be worth being lifted up to 1,200 feet to witness, and such a sight could not fail to leave a long-lasting impression.

Finally, earth tremors, the forerunners of earthquakes, could be felt at the summit of a high tower much more readily than in contact with the earth, and earthquakes might be expected to produce greatly magnified effects at such an elevation; hence a seismometer would find a fitting place at the top of the proposed building.

A great deal might be added on this subject. We have not touched on the astronomical uses of a very high tower, from the greater clearness of the atmosphere at such an altitude. There are also other points of physiological interest that could have been dwelt upon, such as the influence of an altitude of 1,200 feet on respiration, or on the nervous system. It might be remarked that there would be little fear of feeling giddy when looking down form a tower such a height, although the view over a precipice of 1,200 feet would not be unlikely to produce a most unpleasant sensation. This is owing to the fact that there would be high bulwarks round the platform at the top of the tower, giving perfect security; any body may have experienced the absence of giddiness while looking down from the summit of Eiffel's Tower.

Enough has been said, we think, to show that Sir E. Watkins' tower, if constructed, will prove of great use from a scientific point of view, and we trust that due accommodation may be reserved at the top of the tower and down below for instruments and observers.

